# Carbon costs and benefits of France’s biomass energy production targets

**DOI:** 10.1186/s13021-018-0113-5

**Published:** 2018-12-13

**Authors:** Aude Valade, Sebastiaan Luyssaert, Patrick Vallet, Sylvestre Njakou Djomo, Ingride Jesus Van Der Kellen, Valentin Bellassen

**Affiliations:** 1 Institut Pierre Simon Laplace, Place Jussieu 4, 75010 Paris, France; 2 Present Address: Global Ecology Unit CREAF-UAB, Cerdanyola del Vallès, 08193 Catalonia, Spain; 3 Faculty of Science, Free University Amsterdam, VU, 1081 HV Amsterdam, The Netherlands; 4 Irstea, UR EFNO, Domaine des Barres, 45290 Nogent-sur-Vernisson, France; 5 Univ. Grenoble Alpes, Irstea, LESSEM, 38000 Grenoble, France; 6 Department of Agroecology, Aarhus University, Blichers Allé 20, P.O. Box 50, 8830 Tjele, Denmark; 7 INRA, UR1138 BEF, 54280 Nancy, Lorraine France; 8 CESAER, AgroSup Dijon, INRA, Univ. Bourgogne Franche-Comté, 21000 Dijon, France

**Keywords:** Forest management, Renewable energy, Wood harvest, Forest stands, Carbon balance, Forest modelling, Forest growth, Wood-use, Energy substitution, Product substitution

## Abstract

**Background:**

Concern about climate change has motivated France to reduce its reliance on fossil fuel by setting targets for increased biomass-based renewable energy production. This study quantifies the carbon costs and benefits for the French forestry sector in meeting these targets. A forest growth and harvest simulator was developed for French forests using recent forest inventory data, and the wood-use chain was reconstructed from national wood product statistics. We then projected wood production, bioenergy production, and carbon balance for three realistic intensification scenarios and a business-as-usual scenario. These intensification scenarios targeted either overstocked, harvest-delayed or currently actively managed stands.

**Results:**

All three intensification strategies produced 11.6–12.4 million tonnes of oil equivalent per year of wood-based energy by 2026, which corresponds to the target assigned to French wood-energy to meet the EU 2020 renewable energy target. Sustaining this level past 2026 will be challenging, let alone further increasing it. Although energy production targets can be reached, the management intensification required will degrade the near-term carbon balance of the forestry sector, compared to continuing present-day management. Even for the best-performing intensification strategy, i.e., reducing the harvest diameter of actively managed stands, the carbon benefits would only become apparent after 2040. The carbon balance of a strategy putting abandoned forests back into production would only break even by 2055; the carbon balance from increasing thinning in managed but untended stands would not break even within the studied time periods, i.e. 2015–2045 and 2046–2100. Owing to the temporal dynamics in the components of the carbon balance, i.e., the biomass stock in the forest, the carbon stock in wood products, and substitution benefits, the merit order of the examined strategies varies over time.

**Conclusions:**

No single solution was found to improve the carbon balance of the forestry sector by 2040 in a way that also met energy targets. We therefore searched for the intensification scenario that produces energy at the lowest carbon cost. Reducing rotation time of actively managed stands is slightly more efficient than targeting harvest-delayed stands, but in both cases, each unit of energy produced has a carbon cost that only turns into a benefit between 2060 and 2080.

**Electronic supplementary material:**

The online version of this article (10.1186/s13021-018-0113-5) contains supplementary material, which is available to authorized users.

## Background

Since the Kyoto Protocol was signed in 1997, the challenge of limiting and eventually halting the growth in atmospheric CO_2_ concentration has moved to the forefront of the international political agenda, leading to the Paris Agreement in 2015. The ambitious objective of the Paris Agreement is reachable only if energy production becomes decoupled from CO_2_ emissions. At the European level, policymakers have accepted the challenge and made mitigating climate change an objective of the long-term energy strategy [1]. This in turn, is reflected in the national strategies of several European countries, including France.

Currently, nuclear, oil and gas dominate the French energy mix with 43, 30, and 14% of primary consumption [2]. For France, the production of bioenergy is expected to have the largest impact on the CO_2_ emissions by replacing oil and gas. The bioenergy production targets have been set in “the National Forestry and Timber Programme” [3] that focuses on increasing the share of renewable sources of energy. The programme expects to produce an additional 2.3 million tonnes of oil equivalent (Mtoe) per year from biomass for the coming decade, the equivalent of a 25% increase from 2015 [4]. France is thus pushing biomass forward as a major source of renewable energy. This target is expected to be reached through annual mobilization of an extra 12 Mm^3^ of wood [3], which is deemed feasible because the biomass stock of French forests has built up over the past half-century [5, 6].

Diverse processes have contributed to the current biomass stock in French forests: (i) as a result of the industrial revolution, the use of wood as a domestic energy source as well as a building material has collapsed, enabling the forests to recover from centuries of over-use [7]; (ii) advances in agricultural production have resulted in the abandonment of almost 6 Mha of agricultural land in the past century [8] and ambitious reforestation plans launched after World War II (Fonds forestier national; [9]) resulted in the afforestation of around 2 Mha, mainly through softwoods in central France. This large-scale afforestation shifted the national forest age structure towards young forests that will reach maturity in the coming decades [5]; and (iii) 31% of the total area of French forest consists of holdings smaller than 10 hectares [10]. Fragmentation of the ownership reduced the profitability of forest management, resulting in decreasing harvest volume from small forest holdings in recent decades [11]. Following decades in which the annual increment in standing biomass exceeded the annual harvest, French forests are now believed to have the biomass available to supply an additional extra 12 Mm^3^ of yearly wood harvest in a sustainable way [5, 6]. However, the variety of processes that resulted in this biomass accumulation suggests that not all available biomass can be mobilized by a single policy. We account for this complexity in this study (see “Discussion”—different management, different policy).

Furthermore, the scientific basis supporting the contention that meeting bioenergy production targets will result in reduced atmospheric CO_2_ emissions is weak, because it has been shown that the carbon balance of different forest management regimes largely depends on the origin and use of the harvested wood. In the boreal forests of Sweden, a potential increase in carbon sequestration of 6 Mt CO_2_-eq year^−1^ is projected after increasing harvest by 11% [12]. In boreal parts of Finland, however, increasing the harvest levels to reach the biomass energy targets was found to cause a loss of sequestration in those forests; this loss could not be balanced by substituting fossil fuels for wood-based energy and products [13]. In the Pacific Northwest of the USA, the effect of increased harvest rates on carbon sequestration varied from one ecoregion to another but resulted in an increase in carbon emissions for the most productive ecoregions [14]. This study aims to compare the carbon balance of different forest management strategies that would help France to meet its target for wood-based bioenergy production by 2030.

This study has the following objectives: (1) to define explicit intensification strategies, customized for the dominant current forest management approaches, (2) to check which of these intensification strategies meet the bioenergy target by 2030, and (3) quantify the carbon balance of the forest sector when implementing the intensification strategies.

## Results

### Different management styles

The 15.6 Mha of French forests (Table 1) were classified into four management approaches based on their observed characteristics and reported harvest levels (see “Methods”—Management approaches; Calibration Table 2): (1) ‘unexploitable’ forests are neither clearcut nor thinned due to physical constraints on accessibility or exploitability; (2) ‘harvest-delayed’ forests are neither clearcut nor thinned for managerial reasons such as small property size; (3) ‘overstocked’ forests are not regularly thinned but are likely to be clearcut when they reach a sufficient average diameter; and (4) ‘actively managed’ forests are both regularly thinned and clearcut.

**Table 1:** Surface area (km^2^) and standing wood volume (Mm^3^) of the most common tree species in French forests

Species name	Area (km^2^)	Volume (Mm^3^)
*Quercus robur*	21,180	294
*Quercus petraea*	16,140	268
*Quercus pubescens*	14,480	93
*Quercus ilex*	6560	23
*Fagus sylvatica*	14,080	253
*Castanea sativa*	7280	109
*Carpinus betulus*	5500	83
*Fraxinus excelsior*	6530	83
Other hardwood	19,460	420
Total hardwood	111,210	1626
*Pinus pinaster*	10,520	94
*Pinus sylvestris*	9160	91
*Pinus laricio*	1900	Not given
*Pinus nigra*	1800	10
*Pinus halepensis*	2390	17
*Abies alba*	5880	180
*Picea abies*	5670	131
*Pseudotsuga menziesii*	3990	71
Other softwood	3170	296
Total softwood	44,480	890
Total	155,690	2516

**Table 2:** Description of the four management approaches defined in this study

Management approach	Assignation priority	Criteria	Current situation	Management under the reference scenario
Unexploitable	1	Exploitability index is ‘impossible’	Accessibility or exploitability limited by physical constraints	No thinning, no harvest
Harvest-delayed	2	Quadratic mean diameter is above the current practice clearcut diameter defined per species and fertility class	Stands are over-mature	No thinning, no harvest
Overstocked	3	Density index is above the threshold density index defined by species	Stands have not yet reached maturity and their density is too high to be actively managed	No thinning, harvest when clearcut diameter is reached
Actively managed	4	Exploitability index is ‘easy’ to ‘difficult’, quadratic mean diameter and density index are compatible with current management practices defined per species and fertility class	Quadratic mean diameters and stand are in line with present-day forest management for a given species	Thinning, harvest when clearcut diameter is reached

**Fig. 1:**
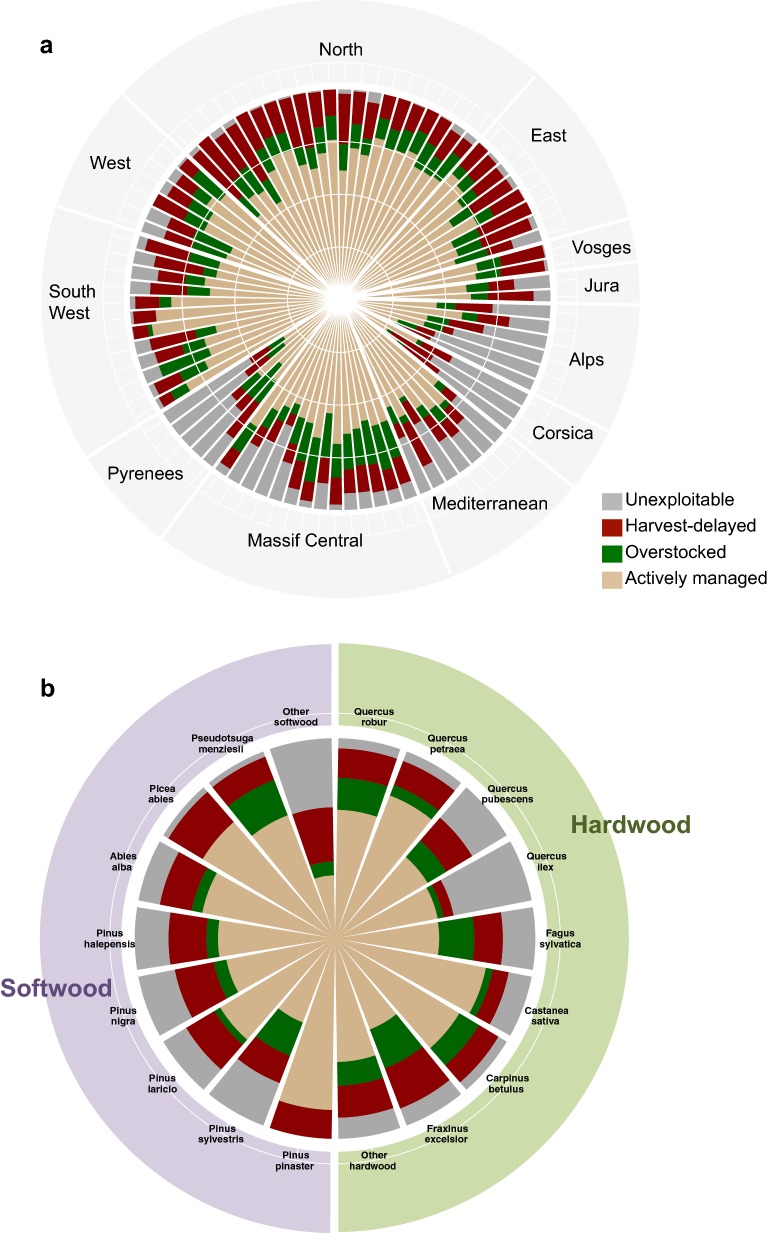
Distribution of management approaches across France. **a** Management distribution by region (individual sectors) grouped by geographical location (region labels) **b** management distribution by wood type and species. Colour fractions of the sectors refer to management categories in each region

At the national scale, 63% of the French forest area was found to be actively managed, 11% unexploitable, 15% harvest-delayed and 11% overstocked; however, large regional differences exist (Fig. 1a for management approaches per silvicultural ecoregion). Most unexploitable forests are located in mountainous areas such as Corsica, the Alps (up to 53.3% of the plots of this region), and the Pyrenees. Harvest-delayed and overstocked forests are relatively evenly distributed across the country, which is consistent with the fact that the drivers for these management approaches are likely to be more related with ownership than with topography or climate. Overall, actively managed forests are the dominant management approach and vary from 27% of the plots in the Corsican mountains to 90% in Les Landes de Gascogne (Fig. 1a).

The type of management also varies with species (Fig. 1b) [4]. Indeed, 86%, 77% and 68% of the French surface cover dominated by Maritime pine (*Pinus pinaster*), spruce (*Picea abies*) and fir (*Abies alba*), respectively, are being actively managed. They are the most managed softwood species in the country. Among the species described in our study, the lowest percentage area being actively managed is the 45% of Scots pine (*Pinus sylvestris*) dominated stands. For the hardwood species, large percentages of oak (*Quercus petraea*, 77%), chestnut (*Castanea sativa*, 77%) and hornbeam (*Carpinus betulus*, 72%) forests are also actively managed.

Whether ‘overstocked’ or ‘harvest-delayed’ forests are more frequent varies from one species to another and from one region to another. For *Quercus robur*, *Fagus sylvatica*, *Carpinus betulus*, and *Pseudotsuga menziesii*, the most frequent type of suboptimal management is ‘overstocked’, meaning that they are likely to be harvested but that the more regular tending of stands is skipped. In contrast, *Fraxinus excelsior*, and three conifer species mainly growing in the southeast of France, *Pinus halepensis*, *Pinus laricio* and *Pinus nigra*, are primarily ‘harvest-delayed’, suggesting that many stands will never be harvested. Finally, where the management is suboptimal for *Quercus pubescens*, *Quercus ilex*, *Castanea sativa*, and *Pinus sylvestris*, this is mostly explained by difficulties with their marketability.

### Meeting the bioenergy target

The French national objectives for energy production from forest biomass foresee an increase from 9.7 Mtoe in 2015 to 11.6–12.4 Mtoe per year in 2026 [3]. Once the target is reached, the plan does not specify whether the 2026 level has to be maintained or whether the annual rate of increase is expected to be sustained over the following decade(s). This lack of clarity in the planning horizon was accounted for by considering two energy production targets: one that was labelled as ‘basic’ and the other that was labelled ‘ambitious’. The basic target prescribes an increase of 1.9 Mtoe between 2016 and 2026. Following 2026, the target for bioenergy production is then held constant at 11.6 Mtoe per year. The ambitious target prescribes an annual increase of 0.25 Mtoe per year between 2016 and 2040.

**Fig. 2:**
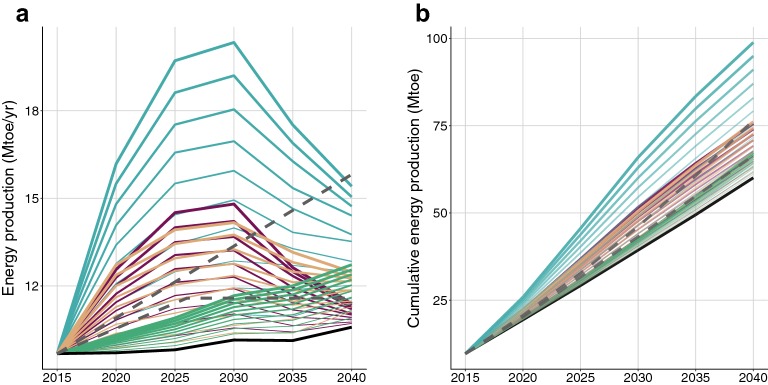
Projected annual energy production (Mtoe/year) from the three intensification scenarios separately (D, Ov, and M) and all three scenarios combined (D + Ov + M) between 2015 and 2040 as **a** yearly or **b** time-cumulative. Blue shows all three scenarios combined (D + Ov + M), brown shows intensification of actively managed sites (M), red shows intensification of harvest-delayed sites (D), green shows intensification of overstocked sites (Ov), and black shows business as usual management of all sites (BaU). The intensification scenarios are presented in section “Methods”—Intensifying forest management and summarized in Table 3. The grey dotted lines show the French national target until 2026 after which time it has not been specified whether the same trend or the same harvest level is to be maintained

Applying a BaU scenario to the forest and wood-use chain increases [3] wood-based biofuel production by 9% by 2040 as an effect of age class structure (Additional file 1). Bioenergy production is thus projected to increase under a BaU scenario, but would still fall 14 to 19% short of meeting the national energy production targeted range in 2025 (Fig. 2; Additional file 1). Meeting the bioenergy target hence requires forest management to be intensified. The intensification scenarios considered in this study match the observed management approaches. Hence, three intensification scenarios were studied (Table 3): thinning currently overstocked stands (labelled as scenario O in Table 3), harvesting currently harvest-delayed stands (labelled as scenario D in Table 3), and decreasing the harvest diameter of actively managed stands (labelled as scenario M in Table 3).

**Table 3:** Description of the intensification scenarios in terms of the management applied to individual stands

Scenario code	Scenario name	Number of simulations	Management of			
			Actively managed plots	Overstocked plots	Harvest-delayed plots	Unexploitable plots
‘BaU’	Reference	1	Harvest at current clearcut diameter; thinning at current thinning ratio	Harvest at current clearcut diameter; no thinning	No harvest; no thinning	No harvest; no thinning
‘O_v_’	Extra mobilization of overstocked forests	10 scenarios applied to 10 to 100% of the overstocked forests	Harvest at current clearcut diameter; thinning at current thinning ratio	Harvest at current clearcut diameter; thinning at current thinning ratio	No harvest; no thinning	No harvest; no thinning
‘D’	Extra mobilization of harvest-delayed forests	10 scenarios applied to 10 to 100% of harvest-delayed forests	Harvest at current clearcut diameter; thinning at current thinning ratio	Harvest at current clearcut diameter; no thinning	Harvest; no thinning	No harvest; no thinning
‘M’	Extra mobilization of actively managed forests	10 scenarios reducing the clearcut diameter by 1 to 10 cm	Clearcut diameter lowered with respect to current clearcut diameter; thinning at current thinning ratio	Harvest at current clearcut diameter; no thinning	No harvest; no thinning	No harvest; no thinning
‘A’	Extra mobilization of all forests	10 scenarios combining respectively the 1st, 2nd, 3rd, 4th, …, 10th scenario of ‘O’, ‘D’ and ‘M’	Clearcut diameter lowered by 1-10 cm with respect to current clearcut diameter; thinning at current thinning ratio	Harvest at current clearcut diameter; thinning at current thinning ratio	Harvest; no thinning	No harvest; no thinning

Over the 30 years of the simulation, the intensification scenarios mobilize an extra 29, 112 or 119 Mm^3^ depending on whether the overstocked, the harvest-delayed, or the actively managed forests are targeted, respectively (Additional file 2: Figure S1). Implementing scenarios that target overstocked forests, provides thinning wood, which is mainly directed to pulp and biofuel. As such it would increase the energy production compared to the reference scenario but would still fall short of reaching the 2026 target (Fig. 2), which would only be reached 3 years later. Note that targeting overstocked forest should not be interpreted as a policy to enhance stand production, because an increase in thinning in the growth and harvest simulator does not lead to an increase of the total biomass production in the simulator.

The two other intensification strategies meet the least ambitious target by 2026, but follow largely different approaches: (a) mobilizing 40% of the harvest-delayed stands, or (b) shortening the rotation length to reduce the clearcut diameter of actively managed stands by an average of 5 cm. Obviously the target could be met by various combinations of all three intensification scenarios, for example, shortening the rotation length to reduce the clearcut diameter of actively managed stands by an average of 2 cm, mobilizing 20% of the harvest-delayed stands, and at the same time thinning 20% of the overstocked stands (Fig. 2).

The most ambitious bioenergy target can also be reached by 2026, for example, by decreasing the harvest diameter by 7 cm and thus shortening the rotation length of actively managed forests, or by mobilizing 60% of the harvest-delayed stands. For the latter, the transient character of mobilizing harvest-delayed stands would result in no longer meeting the target after 2030 even though the bioenergy production exceeded the yearly target in the previous decade (Fig. 2a).

Note that these scenarios do not allow to sustain the target after 2026. In order to reach the most ambitious target sustainably over 2026–2040, it is necessary to combine more intensive scenarios—such as the mobilization of 100% of harvest-delayed stands or a 10 cm reduction of clearcut diameters of actively managed stands—with the possibility for a delayed use of the biomass several years after harvest took place (Fig. 2b). While storing wood may not be realistic, this possibility for a delayed use of biomass can be interpreted more realistically as a proxy for a more gradual implementation of the intensification scenarios.

### Carbon balance of intensified management scenarios

Depending on the intensification scenario, the properties of the mobilized biomass are projected to differ in terms of tree species and the relative contributions of thinnings versus clearcuts to the total harvest. Consequently, different intensification scenarios result in different amounts of wood products and different levels of energy production.

**Fig. 3:**
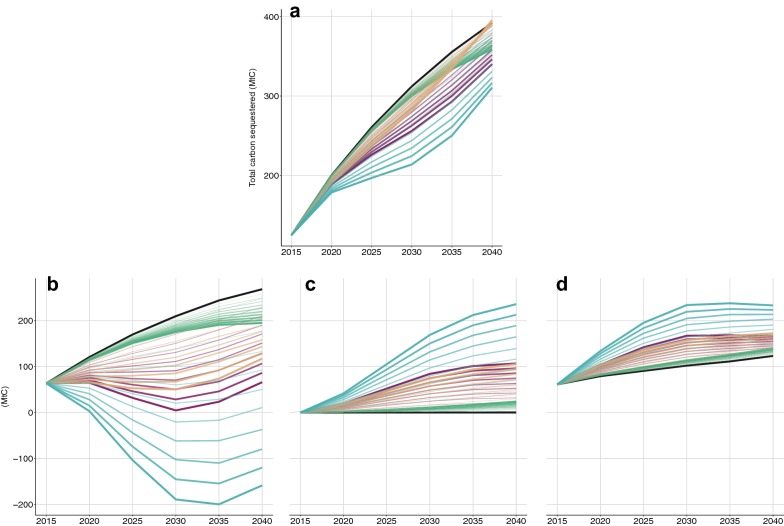
Projected evolution of the French forest sector carbon balance until 2040 and its main components for the three intensification scenarios separately (D, O_v_, and M) and all three scenarios combined (D + O_v_ + M) between 2010 and 2040. **a** Total carbon sink (Tg C), **b** carbon sink in the forest or in situ sink (Tg C), **b** emission savings through energy substitution (Tg C), and **c** carbon sink in wood products (Tg C). Blue shows all three scenarios combined (D + O_v_ + M), brown shows intensification of actively managed sites (M), red shows intensification of harvest-delayed sites (D), green shows intensification of overstocked sites (O_v_), and black shows business as usual management of all sites (BaU). The intensification scenarios are presented in section “Methods”—Intensifying forest management and summarized in Table 3

Although the simulation shows that management intensification could help France meet its target for wood-based bioenergy production, none of these intensification scenarios would result in a higher atmospheric CO_2_ sequestration than the reference scenario by 2040 (Fig. 3a). Intensification does indeed affect the three components of the forest sector carbon balance: the in situ carbon stock (Fig. 3b), the carbon stock in the wood products pool (Fig. 3d), and carbon emissions avoided by substitution (Table 4; Fig. 3c).

**Table 4:** Substitution coefficients and lifetime expectancy of wood products

Wood products	Lifetime expectancy		Substitution coefficient	
	Value (years)	References	tC/tC	Reference
Timber	50	[92]	1.2	FCBA (pers.comm.)
Paper (44% of pulp and paper)	4		0	FCBA (pers.comm.)
Pulp (56% of pulp and paper)			0.53	FCBA (pers.comm.)
Energy	1.7	[97]	0.5	In substitution to: gas at 81%, oil at 15%, coal at 3%, electricity at 0.4% and GPL at 0.4% [98]

All three scenarios affect the carbon pools in the same direction but with different intensities. Increasing harvest decreases the carbon stored in the forest, and increases both the carbon stored in the wood products pool and in the apparent stock from energy and product substitution.

In the first years following intensification of the management of harvest-delayed and actively-managed stands, the in situ carbon stock will decrease rapidly and the decrease will not be compensated for by the increase in wood-based carbon stock and energy through product substitutions. The result is a decrease in carbon sequestration of 31 t C/year from harvest-delayed stands and 20 t C/year from actively-managed stands compared to BaU by 2025 (Fig. 3a). The mobilization of harvest-delayed forests yields the largest and fastest decrease in the in situ carbon stock with the loss of 75% of the carbon stored under the BaU scenario by 2040. The gain in the apparent stock from substitution would be 106 Tg C by 2040 (Fig. 3c), thus compensating for only 52% of the carbon stock lost in the forest. Increased carbon stock in wood products would compensate for 21% of the carbon stock lost in the forest.

The intensification scenario that foresees a shortening of the average rotation lengths of actively managed forests results in a different carbon balance. The changes in wood product pools and substitution are very close to the ones obtained for the harvest-delayed strategy; however, the change in the in situ carbon stock shows a faster recovery of the stock. Hence, by 2040 the most intense scenario reducing harvest diameters by 10 cm, decreases carbon stock in the forest to 52% of BaU. Nevertheless, this loss is compensated for by the gain in carbon stock in wood products and the apparent storage from substitution. For this intensification scenario, the net carbon balance in 2040 comes close to that of BaU (Fig. 3a).

Intensifying the management of overstocked forests will reduce the in situ carbon stock (Fig. 3b). This reduction will be compensated for neither by carbon storage in wood products (Fig. 3c) nor by the substitution of fossil fuel-based energy provision by wood-based energy provision (Table 4; Fig. 3d). Consequently, the carbon benefit decreases in proportion to the extra mobilization (Fig. 3a)—a relationship that was also observed in Finland for a range of harvest ratios applied at the national level [15]. Compared to other intensification strategies, thinning overstocked stands yields a better carbon balance than scenarios targeting mature forest in the first decade because it causes less of a reduction in their in situ carbon stock (Fig. 3a, b). In the long term, however, because there is no feedback of thinning on stand-level biomass production, limited feedback of thinning on mortality (see “Methods”) and because of low substitution and low life expectancies of wood-products derived from thinning (Table 4; Fig. 3c, d), thinning overstocked stands becomes less beneficial than intensifying the management of actively managed stands and by 2045 it even becomes less beneficial than intensifying the management of harvest-delayed stands (Additional file 3: Figure S2).

### Transient biomass mobilization

Intensification scenarios targeting harvest-delayed and actively managed stands rely heavily on biomass that has accumulated over the past decades to century that is progressively being mobilized. The transient increase in harvest is evident in Figs. 2a and 3b from the bell-shaped trajectories. Once this stock has been consumed, a new equilibrium will emerge between harvest and growth. Projections for a longer time period (Additional file 3: Figure S2) show the new equilibrium that will be reached after the legacy biomass stock is consumed. Note that this new harvest equilibrium is above the BaU level.

**Fig. 4:**
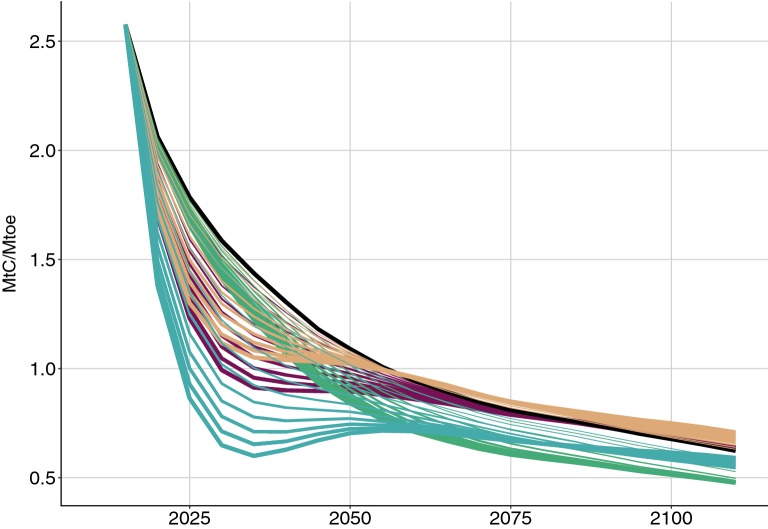
Carbon efficiencies of energy production (Mt C sequestered per Mtoe produced) for the three intensification scenarios separately (D, O_v_, and M) and all three scenarios combined (D + O_v_ + M) between 2010 and 2115. Carbon efficiencies were calculated as cumulated total carbon balance (Mt C) divided by cumulated energy production (Mtoe). Blue shows all three scenarios combined (D + O_v_ + M), brown shows intensification of actively managed sites (M), red shows intensification of harvest-delayed sites (D), green shows intensification of overstocked sites (O_v_), and black shows business as usual management of all sites (BaU). The intensification scenarios are presented in section “Methods”—Intensifying forest management and summarized in Table 3

In the first years of management intensification, the mobilization of accumulated biomass in the actively managed and harvest-delayed forests make the energy they produce much less carbon-efficient than the energy produced under the BaU scenario (Fig. 4a). This carbon efficiency gap persists until 2045 and 2055 respectively, when previously harvested stands will have regrown and the substitution benefits have accumulated (Fig. 3d).

## Discussion

### Different management styles require different policies

Close to 75% of French forests are privately owned and substantial differences in management approaches are observed (Fig. 1a, b), i.e., unexploitable, harvest-delayed, overstocked and actively managed forest (Table 2). Over 60% of private owners own less than 15% of the forest because their individual holding is less than 4 ha [16]. French law does not require a formal management plan for small holdings [17]. Hence, one consequence of the fragmented ownership is that a formal management plan been made for only 48% of the French forest area [18] compared to 66% at the European scale [19].

Despite such management diversity, we are not aware of studies that differentiate their intensification scenarios based on the current management. Recent studies in France with nationwide management scenarios [6, 20] uniformly increased harvest rates for species and climate-specific study units. Yet, differences in management approach may partly reflect underlying differences in the forest owners’ attitudes and expectations such that policies targeting forest under one specific management approach will not necessarily succeed in mobilizing wood from the other management approaches [21, 22].

A policy subsidizing thinning or incentivizing the development of management plans would most likely target the overstocked forests whose owners are motivated by financial profits as is reflected by the fact that they do harvest eventually [23, 24]. These owners, however, generally lack the skills or motivation to plan for forest thinning and other management measures. A policy targeting downstream parts of the wood-sector, for example through end-product price subsidies, would most likely stimulate the mobilization of the forests that are actively managed, because these owners have a management plan and could adapt their planning to the market.

Targeting harvest-delayed forests and their owners could be more challenging as owners appear not to be motivated by financial gains through wood selling as reflected by the fact that they did not seize the opportunity to harvest their forest. Mobilizing wood from harvest-delayed forests will likely require developing a strategy based on information, regrouping of small holdings [25, 26], guidance and services. Finally, biomass mobilization from unexploitable forests—not explored in this study—will require large-scale development of infrastructure [27] such as extraction rides, bridges, and/or investments in harvesting methods previously unused in France, such as cable harvesting [28].

### Carbon costs of energy production

The carbon balances described above all mobilize different amounts of wood, produce different amounts of energy, and retain different amounts of carbon in the forest. Given that wood harvest and subsequent bioenergy production differ between scenarios, comparing the carbon balance of scenarios goes back to comparing different levels of service provision. Let us consider that the challenge for future forest management is not simply to produce as much energy as possible, nor to produce the largest sink as possible, but instead to serve society at the lowest possible carbon cost. The optimal intensification scenario is then the scenario that produces bioenergy at the lowest carbon cost. Hence, this trade-off was calculated by normalizing the carbon balance by the total energy produced, defining the carbon efficiency of biomass energy, following an approach similar to the previously defined relative carbon indicator [29] and carbon neutrality factor [30, 31].

For BaU the carbon efficiency of biomass energy production decreases reflecting the forest age dynamics and decrease in in situ carbon sequestration as stands reach maturity. Although BaU and decreasing the harvest diameter by 10 cm (M-10) will have the same overall carbon balance by 2040 (Fig. 3a), their carbon efficiencies of biomass energy production differ: 1.04 Mt C sequestered per Mtoe of energy produced for M-10 versus 1.31 Mt C sequestered per Mtoe produced under BaU (Fig. 4). Similarly, mobilizing 20% of the harvest-delayed forests (D-2), decreasing the harvest diameter by 2 cm (M-2), and mobilizing 70% of the overstocked forests (O_v_-7) all produce between 290 and 292 Mm^3^ of wood over 30 years. Nevertheless these three intensification scenarios have very different carbon efficiencies in 2040, with 1.21 Mt C sequestered per Mtoe for D-2, 1.24 Mt C sequestered per Mtoe for M-2, and 1.12 Mt C sequestered per Mtoe energy produced for O_v_-7. Figure 4 compares these figures to the 1.31 Mt C sequestered for each Mtoe of biomass energy produced under the reference scenario.

Differences between the carbon sequestration potentials per unit of energy produced by the different management strategies thus reveals that not all units of bioenergy have the same carbon mitigation potential which itself varies with time (Fig. 4) in line with previous studies. Zanchi et al. [31] compared the carbon balance of energy biomass from increased removals in a managed forest, both from increased fellings and from extracting residues. They found that the carbon benefits of the different strategies after 100 years vary widely from the increased fellings, for which the carbon balance is more negative than the reference, to the use of residues, for which emissions are 76–85% lower than the reference scenario. Similar conclusions were drawn by McKechnie et al. [32], who compared the carbon balance of four sources of biomass energy and their fossil fuel alternatives. Over 100 years, using residues for energy showed larger carbon benefits than using standing trees, whatever the amount of fossil fuel replaced.

### Carbon parity time of intensified management scenarios

Only after 2040 is wood energy produced under intensified scenarios for actively managed forests or in harvest-delayed forest likely to result in lower atmospheric CO_2_ emissions compared to the BaU (Fig. 3a, 4, Additional file 3: Figure S2). Producing energy from increasing harvest in actively managed stands has a carbon cost over 25 years peaking in 2025 with 0.6 less t C sequestered per ton of oil equivalent energy produced compared with BaU. From 2035 onwards, energy production comes with carbon benefits with respect to BaU management (Fig. 4a), but it will take another 10 years before the debt accumulated in the first 25 years is compensated for and the benefits can be observed in the atmosphere (Fig. 4b).

For energy produced from harvest-delayed stands, the carbon cost per unit of energy produced peaks in 2025 with 0.7 additional t C emitted per toe produced compared with BaU, the carbon benefits would emerge in 2040 (Fig. 4a) and it would take another 15 years for the additional emissions to be compensated for (Fig. 4b). The longer time frame needed to realize the benefits for harvest-delayed intensification is due to the fact that the decrease in the in situ carbon stock is larger when targeting harvest-delayed rather than actively managed stands whereas both scenarios result in similar substitution benefits (Fig. 3b).

In turn, for the strategy targeting overstocked forests, directing thinning to energy production instead of directing harvest to timber has a carbon cost that is apparent in the crossing with other intensification strategies (Fig. 4) since before 2040 the energy produced from overstocked forests is already more expensive in terms of carbon emissions than the energy from actively managed and harvest-delayed stands. Indeed, energy production from targeting the overstocked forest would never result in additional carbon sequestration compared to the BaU scenario. This feature comes from the weak correlation in the inventory data between stand density and mortality: mortality is only proportional to standing volume (not shown). Accordingly, the intuition that thinning overstocked forests partly replaces natural mortality with harvest is not quantitatively borne out in our empirical model.

When targeting mature forests, carbon benefits take time to materialize (Figs. 3, 4; [33] given the combination of factors playing on two scales. On the one hand, at short time scales, recently clearcut stands have low biological production [34], not all biomass removed is transferred to wood product pools due to management operation losses [35] and to the use of wood for short-lived products, and compared to fossil fuel, a larger amount of wood-based carbon is needed to produce the same amount of energy. On the other hand, at a longer time scale, shortening the rotation length increases the overall biological production due to a larger proportion of younger forests becoming more productive than the older forests they are replacing [34] and substitution benefits accumulate over time. The long-term processes hence pay back the carbon debt generated from the short-term ones. The relative weights of these different processes determine if and when intensified harvest will yield carbon benefits.

The carbon debt and parity time have been the object of an extensive body of research, often focused on the single energy use of forest harvest and using a large variety of assumptions and methodologies leading to an even larger range of estimated parity times. As shown by Lamers and Junginger [36], Mitchell et al. [37], Holtsmark [38], or Bentsen et al. [39], the parity time depends mainly on the assumptions on the initial state of the forest, the forest growth rate and management practice. The assumptions used in the present study are sought to be as realistic as possible within the French context.

Here the carbon parity time is analysed at the national scale with a forest inventory-based dynamic forest model that considers the full wood products transformation chain and thus accounts for: multiple harvests, as suggested by Holtsmark [38]; for cascading and end-of-life of wood products, as highlighted by Geng et al. [40]; and the growth in the reference scenario, as advocated by Holtsmark [41]. Our finding that the carbon parity time is longer when targeting harvest-delayed forest than when targeting actively managed forests is consistent with the energy-only sensitivity analysis carried out by Laganière et al. [42] who find parity times longer for stands with slower growing trees. Our estimates of carbon parity times are in the lower range of the values reviewed by Bentsen [39] and Lamers and Junginger [36]. This must be the consequence of the high fraction of harvest used for long-lived timber products when most reviewed studies only consider the use of harvest for energy, and of the high level of cascading in the wood-use chain that leads to the same unit of harvest substituting for multiple alternative products.

In our opinion the search for the carbon parity time of increased harvest per unit energy is highly relevant for policymakers because it effectively considers the carbon stocks in the atmosphere, the in situ carbon stocks as well as the production of wood-based energy. Following these considerations, the carbon parity time of intensifying actively managed forest is 15 years lower than the carbon parity time of targeting harvest-delayed ones (Additional file 3: Figure S2). Targeting currently managed forests and intensifying their management thus appears as a more promising approach. Indeed, this approach could be seen as the forest-based equivalent of a concept known in agricultural science as land sparing [43–46]. The land-sparing concept separates forest for wood and fibre production from forest for conservation. With proper management high production levels can be realised in the managed parts of the forest, enabling the protection of the remaining forest. Note however, that forest production may depend on biodiversity [47], which suggests that land-sparing approaches should be carefully designed to qualify as truly sustainable forest management [46].

To the contrary, once a forest has been left to accumulate a substantial biomass stock, as is the case for the harvest-delayed forests in France, going back to a typical management approach comes at a very high carbon cost. If it is considered essential that the harvest-delayed forests are taken back into management, management that avoids the sudden release of the carbon stock (‘conservation management’) should be developed. Taking harvest-delayed forests back into production would relate to a land-sharing approach, which is centred around the integration of diverse functions, including biodiversity conservation, and wood production, in the same forest [46].

### Limitations

Uneven-aged stands and stands for which no stand structure was reported in the inventory each cover 5% of French forests. Respectively 36 and 40% of these stands are classified as unexploitable or harvest-delayed but when management applies, these stands are respectively managed as even-aged high stands and coppice. This assumption is expected to have little impact on the growth and harvest estimates due to the low area covered.

A source of the model lack of fit, for all variables that were evaluated (Additional file 4: Figure S3, Additional file 5: Figure S4), is the statistical weight applied to individual plots when upscaling the plot data to the regional and subsequently national level. The National Forest Inventory agency (NFI) acknowledges that in a few homogeneous regions, they take smaller samples [48]. Accordingly, this undersampling is corrected by applying statistical weight to the plots located in these regions [49]. These statistical weights, however, are not publicly available. Estimates of standing volumes referred to as observations were extracted from an NFI report [50] and thus make use of these weights, whereas the simulated standing volume applied equal weights to all plots to derive the per unit area variables that are then upscaled with the reported areas for each species per region.

The growth and harvest simulator, which is the methodological basis of the study, was set up for single-species stands (see “Methods”—Growth and harvest simulator), hence, mixed stands in the inventory data were treated as homogeneous stands of the dominant tree species in the mixture. This homogenization of assigning mixed stands to just one species leads to an overestimation of the harvest of the dominant species, offset by an underestimation of the harvest of all other species (Additional file 4: Figure S3e). Species mixing is implicitly represented in the stand growth as the volume increment growth equation was derived from whole plot growth estimates.

The growth and harvest simulator, being an inventory-based empirical model, assumes the recent growing conditions are maintained and focuses on the interplay between forest management and the three carbon pools of the forestry sector, i.e., the in situ carbon stock in forests, the carbon stock in wood products and the apparent carbon stock in product and energy substitution, and their changes with age and species distributions. As such, the effect of recent disturbances on in situ carbon stocks are assumed to remain constant but neither future effects of climate change on forest growth [51] nor the feedback of management on soil nutrients [52, 53] were accounted for.

Previous studies suggest a minor effect of forest management on soil carbon, with the exception of whole tree—including stump—removal [54]. In the context of climate change, Schelhaas et al. [55] describes the increase of extreme events in the recent years (1950–2000). Under future climate, it is unlikely that the volume of wood burnt in forest fire would increase drastically because of investments in fire prevention and fighting. There is a risk that the frequency and intensity of damages from storms, pests and diseases increases in the coming decades. While these limitations are unlikely to impair our conclusions for the first 30 years of simulation, the assumption of constant growing conditions cannot be expected to remain valid beyond that. Climate change itself, as well as its expected effects on forest growth, are still the object of large uncertainties which, for time horizons over 50 years, dominate the variance of the forest sector carbon balance [56]. The interaction of management and the climatic response of forests is beyond the scope of this study and was therefore not considered in the growth and harvest simulator.

This study assumed that the extra wood mobilized in each intensification scenario will be directed in the same ratios as today to timber, pulp and paper, and energy production hence ignoring the socioeconomic dynamics of the forest sector. This approach reflects the assumption that over the next 30 years, economic or political change will be sufficiently smooth to avoid high quality wood being used for energy production rather than in construction. This assumption effectively constrains the potential bioenergy production from French forests since increasing the harvest of mature trees only increases energy production along the wood processing chain of the timber and pulp wood products through increased sawing residues and end-of-life recycling and burning. Thinning thus contributes more strongly to energy production than clearcuts because the quality and dimensions of the thinned wood are, at present, not high enough for direct use in timber production [57, 58]. Accounting for changes in wood demand, wood price, technological efficiency, consumer behaviour, substitution potential of products and energy calls for complex socioeconomic scenarios and modelling of feedback between processes that fall out of the scope of this study.

## Conclusions

We have quantified the changes in the carbon balance of the French forestry sector following implementation of intensification scenarios. The novelty of our approach was linking the intensification scenarios to present-day management approaches. This paired approach towards intensification reflects the reality where mobilizing wood will require incentives that are likely to trigger actions from one kind of owner, but not necessarily from all of them. The description of the economic and political tools to realize these scenarios is outside the scope of this study.

Using different management measures to mobilize similar amount of wood from forest currently under different management, i.e., overstocked, harvest-delayed or actively managed, was found to lead to substantial differences in the potential to produce wood-based energy, as well as to mitigate CO_2_ emissions from fossil fuel burning. This finding suggests the need for policymakers to be more precise in specifying from which forests the extra wood will be extracted. It should be noted that differences in the physical environment as well as differences in owners’ attitudes may require very different approaches to extract wood from forests that are currently under different management regimes.

The economic rationale behind our findings is that high quality wood is used for applications with a high added value and low quality wood is used for applications with little added value, and the current wood transformation flows remain valid over the coming decades. If this reasoning is accepted, several realistic intensification scenarios exist to enable France to reach it’s bioenergy target for 2026. Any increase in wood energy use beyond that target may rapidly become unrealistic due to the forests’ dependence on the in situ carbon stock built-up over the past decades and centuries rather than depending on the actual production of French forests.

Differences between the carbon sequestration potentials per unit of energy produced of different intensification scenarios revealed that not all units of bioenergy have the same carbon mitigation potential, which itself varies with time. In terms of time that will be required to compensate for the extra carbon emissions made during the first decades of the intensification, the most favourable policies are those targeting forests that are currently actively managed followed by policies targeting forests for which the harvest has been delayed.

Recent studies suggest that managing forests for their carbon balance may have adverse effects on forest structure when the aim of the forest management is to contribute to climate change mitigation [59–62]. This concern is fuelled by the observation that forest management affects not only carbon emissions and sequestration, but all aspects of the forest’s interaction with climate including water exchanges, radiation interception, and volatile organic compound emissions [63–68].

There are several potential reasons for intensifying forest management in France: developing the rural economy [7, 69], reducing the trade imbalance [70], adapting stands to climate change by, for example, preventive thinning to reduce the risk of forest fires [71] or by thinning to reduce the water use by forests [72–74]. Nevertheless, our analysis refutes the idea that intensifying forest management in France will reduce the carbon emissions of the forestry sector before 2045 and as such help the country to meet its commitments towards the Paris Agreement. Well-designed intensification may have this effect, but it is not guaranteed to result in emission saving after 2045, and until then stricter emission reduction would be required by other sectors to compensate for the transient increase in emissions from forestry.

## Methods

### Methods overview

**Fig. 5:**
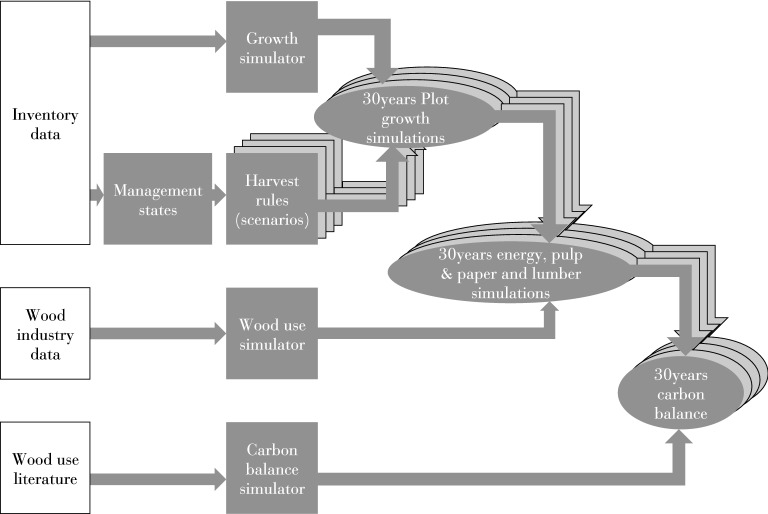
Flowchart of data processing in this study. The NFI data, the growth and harvest simulator and the wood-use simulator were used to obtain projections of the future carbon balance of the French forest sector under different scenarios of forest management intensification

A growth and harvest simulator was developed for the purpose of this study (see “Methods”—Growth and harvest simulator). The initial conditions of the forest and the growth equations of the simulator were parameterized by making use of the French National Forest Inventory Data (hereafter called NFI) (Fig. 5) for the years 2008–2012 [50].

Each plot of the IFN dataset was assumed to be homogeneous in species and stand structure, be it high stand or coppice. Density, fertility and exploitability indices were estimated from the IFN data (see “Characterizing stands using the French National Inventory data” section), used as proxies for the current management state (see “Management approaches” section), and used to build the growth and harvest simulator based on empirical growth relationships derived for volume and diameter (see “Methods”—Growth and harvest simulator).

The simulator assumes that the wood market is supply-driven: stands are harvested when they are mature, irrespective of the demand and wood price. Such a simplification is justified by the relative inelasticity of wood supply [75] and obviates the need to include wood demand or wood price scenarios. The simulated harvest at the first model time-step, 2015, was calibrated to match the nationwide total harvest per species [50], by adjusting the thresholds used to define the different management approaches, i.e., harvest diameter and thinning ratio (see “Methods” Calibration). By combining the descriptions of management and growth, and assuming constant environmental conditions, the evolution of French forest was simulated for a set of diverse but realistic management scenarios (Fig. 5). Furthermore, the wood-use chain was reconstructed from wood industry data. Forest growth, wood use, lifetime, and substitution potential of all wood products were accounted for in the carbon balance of the set of management scenarios which represented different intensification approaches.

The growth and harvest simulator was ran for a Business as Usual (BaU) scenario for all of France between 2010 and 2115 with 2040–2115 results solely used to determine the carbon parity time (see “Discussion”—Carbon parity time of intensified management scenarios). Subsequently, simulated annual harvests were fed into the French wood-use chain to project the stock of wood products as well as the energy production and emissions from wood use.

In this study, BaU represents a scenario in which no political action is taken, and no economic incentive exists, to increase wood harvest above present-day levels and the transformation industry is able to adapt its capacity to absorb the variation in wood supply. The BaU scenario thus assumed that no change in practice occurs, either in harvesting levels or in wood use. In the future, forests are thus assumed to be managed as they are today (see “Discussion”—Different management, different policy) and the harvested wood is assumed to be transformed in the same proportions into timber, pulp, and paper and energy with the same transformation efficiencies as today—whatever the simulated wood supply (see “Methods”—Wood use modelling). Changes to this assumption would require socioeconomic hypotheses that would impair the understanding of the processes driving the carbon balance and fall beyond the scope of this study.

### Characterizing stands using the French National Inventory data

Every year a tenth of a 1 × 2 km grid over France is visited by IFN, resulting in the inventory of around 6500 to 7000 forest plots a year. On each inventory point, four circular plots of decreasing radius are established to measure and observe different variables with an appropriate level of detail [49]. Each stand is described in terms of the physical properties of the terrain, e.g., topography and soil properties, and its vegetation characteristics. Mainly tree-level and stand-level measurements were used in this study.

This study explicitly analysed the data of the 14 tree species which comprise 85% of the total area of forest in France (Table 1). Inventory plots for which the main species was not one of these 14 selected species, were pooled in the categories ‘Other hardwood’ or ‘Other softwood’. Some further simplification was made when upscaling the IFN data from the plot to the regional or national level. The IFN methodology under-samples a few homogeneous regions [49]. When upscaling the data, the effect of this sampling bias needs to be corrected by weighting plots by a statistical weight that describes their representativeness in terms of forest area. These statistical weights, however, are not publicly available [76]. Hence, an equal weight for all plots was used when averaging the variables within each species-region combination. The NFI-reported areas of each species-region combination is then used to upscale the data to the national scale.

#### Site index

Site index is an integrated metric commonly used in forestry to evaluate the quality of a site for tree growth [77]. It usually takes the form of a height at a given reference age, and is usually set to 100 years, as is the case in this study. From the IFN measurements of tree-level age and height, we reconstructed the stand-level variables of average stand age (average of the measured ages) and dominant height (*h*0; average height of the 100 trees with largest diameters) for each species. A Hossfeld II type equation [78]—a sigmoidal function commonly used to model biological growth—was fitted to the dominant height-age data, and used as a reference to describe the dominant height (h0; m) as a function of age [79]:

1 $$\begin{array}{*{20}c} {Guidecurve{:}\, h_{0\,guide} \left( {age} \right) = \frac{{a_{guide} }}{{1 + \left( {\frac{b}{age}} \right)^{c} }} = a_{guide} *f\left( {age} \right)} \\ {h_{0\,guide} \left( {100} \right) = H100_{guide} = a_{guide} *f\left( {100} \right)} \\ {\frac{{H100_{guide} }}{{h_{0\,guide} \left( {age} \right)}} = \frac{{f\left( {100} \right)}}{{f\left( {age} \right)}}} \\ \end{array}$$

where *a*, *b*, and *c* are fitted coefficients, *h*0 (m) and *age* (years) are the plot dominant height and age, respectively (Additional file 6: Table S3). Assuming a constant shape across fertility classes (constant parameters b and c, hence same function *f*) the ratio between the site index and the height at age t (years) are constant across fertility indices [80]:

2 $$\frac{{H100_{plot} }}{{h_{0\,plot} \left( {age} \right)}} = \frac{{H100_{guide} }}{{h_{0\,guide} \left( {age} \right)}}$$

where *H*100_plot_ (m) is the dominant height at an age of 100 years sought for a given plot, *h*_0plot_(*age*) (m) is the observed height for the current age of the plot, *H100*_guide_ (m) is the dominant height calculated from the guide curve for an age of 100 years, and *h*_0guide_(*age*) (m) is the dominant height calculated from the reference curve for the age of the considered plot. H100 was used as the site index and the fertility indices for each species were aggregated into fertility classes for which the limits were defined by the observed quantiles of the distribution of site indices for each species (Additional file 7: Figure S5).

#### Density index

The relative density index (DI) is commonly used to quantify management intensity [81]. It has been defined as the ratio of the number of stems in a stand and the expected number of stems for a stand of the same mean diameter [82] under self-thinning [83]. For each species, the boundary of the log–log relationship between the observed quadratic mean diameter and the stand’s number of stems for stands with age between the 10th and 90th percentiles was fitted to quantify the self-thinning relationship [84]. Owing to the presence of unmanaged stands in French forests, some of the observations can be expected to be close to self-thinning, which is required for this approach to be valid.

The DI is then calculated as:

3 $$DI = \frac{N}{{N_{max} }} = \frac{N}{{e^{g} Dg^{h} }}$$

where *DI* is the density index (unitless) for a stand characterized by its number of stems *N* (trees ha^−1^) and quadratic mean diameter *Dg* (cm) as calculated from the inventory’s circumference data. *N*_*max*_ (stems ha^−1^) is the maximum number of trees with quadratic mean diameter *Dg* according to the fitted self-thinning relationship. Parameters *g* and *h* are fitted to define the self-thinning function as log(*N*) = *g *+ *h* log(*Dg*). An example of a fitted species-specific self-thinning relationship is shown in Additional file 8: Figure S6 and parameters of the self-thinning relationships are shown in Additional file 6: Table S3.

#### Exploitability index

Following the methodology established by IGN [49], plot-level records of terrain slope, ruggedness, distance to forest ride, carrying capacity of the ground for machinery were used to define four levels of exploitability: very easy, easy, difficult, impossible. The details of the categories are shown in Additional file 9: Table S1.

### Management approaches

Plots were classified as one of four management approaches based on their physical and biological characteristics: quadratic mean diameter, fertility index, relative density index, and exploitability index. The use of the exploitability index as a physical constraint builds on the assumption that the stands which were easier to harvest and thin, are more likely to have been harvested. However, depending on the wood price, targeting stands with a lower exploitability index could be justifiable. Price dependency may have contributed to the observation that *P. pinaster* stands were managed irrespective of their exploitability indices. Harvest and thinning of all other species started with the exploitability index set as ‘difficult to access’.

Discontinuity in the statistical distribution of the diameter was used to determine the expected clearcut diameter for each species and each fertility class. The discontinuity was identified using a segmented regression approach. The rationale for this is the assumption that for a given species, under idealized management and an even age-class distribution, all diameter classes below the harvest diameter would have a similar number of stands whereas no stand would have a mean diameter above the harvest diameter. Subsequently, the clearcut diameters calculated with this method served as a prior and were adjusted during calibration of the harvested wood volume per species, as reported by IFN for the year 2010 [50]. Also, for each species, a density threshold was set based on *DI* distributions with a segmented regression approach similar to the one used for the diameters. Subsequently, the final density threshold was determined through calibration of the harvested wood volume per species, as reported by IFN for the year 2010 [50].

Each plot is assigned to only one of the four management approaches by following an assignment order. First, plots with too low an exploitability index were classified as unexploitable. Then, the remaining plots with diameters above the calibrated expected quadratic mean diameter were referred to as ‘harvest-delayed’ and assumed not to be harvested for ownership reasons, for example, fragmentation of properties or disinterest of the owner or for conservation. Under present management, or Business as Usual, these plots are neither thinned nor harvested. Then, plots with densities above the final DI threshold were referred to as overstocked. In a Business as Usual scenario, these plots will not be thinned but will be harvested. Finally, the remaining forest with diameters, density and exploitability not matching any of the above criteria, were classified as actively managed. For *P. pinaster* and *Picea abies* stands, the harvest statistics were best matched when no threshold was set either on stocking density or on exploitability index.

### Growth and harvest simulator

#### Forest growth

The growth and harvest simulator calculates the evolution of each plot in the inventory, based on its species, density and fertility characteristics. The 5-year radius increment and the 5-year volume increment at the stand-level are the two key variables of the growth and harvest simulator. The incremental variables are modelled, rather than absolute variables, because the initial distributions of the volume and diameter are preserved. Volume and radius increment are independently simulated (Eqs. 4 and 5, respectively) as a function of stand age, its growing stock and stand fertility. The structure of the equations used in the growth and harvest simulator are commonly used in the forest modelling community [80].

4 $${ \log }\left( {IV_{stand} } \right) = a_{H100} + {\text{a}}_{DI} \cdot { \log }\left( {DI} \right) + {\text{a}}_{age} \cdot { \log }\left( {age} \right) + \varepsilon$$

5 $$log\left( {Ir_{stand} } \right) = b_{H100} + b_{DI} \cdot log\left( {DI} \right) + b_{age} \cdot log\left( {age} \right) + \varepsilon$$

where *IV* is the 5-year volume increment (m^3^ ha^−1^), *age* is the plot age (years), *DI* is the density index (unitless), *ε* is the residual error term, *a* and *b* the regression coefficients for the log-transformed variables [80].

Ultimately, Eqs. 4 and 5 were to be used to simulate the future productivity of French forests under different intensification scenarios and BaU. This, however, required that the parameters of this set of equations, i.e., *a*_*H100*_, a_DI_, a_age_, b_*H100*_, b_DI_, b_age_ were fitted first on inventory-based plot observations. As the stand-level volume and above-bark diameter increments are not reported in the inventory, *IV*_*stand*_ and *Ir*_*stand*_ were estimated from the plot measurements. The subsequent paragraphs of this section detail how *IV*_*stand*_ and *Ir*_*stand*_ were calculated.

#### Derivation of stand-level volume and radius increment

The modelling at the stand level required making an assumption on stand composition. Each stand in the national inventory data was assumed to be homogeneous in terms of its species composition. The species for which the age was recorded in the NFI, was considered the main tree species of the stand and was used in the species-specific equations of the growth and harvest simulator.

The stand-level volume increment (*IV*_*stand*_; m^3^) is calculated as the weighted sum (w_tree_) of the volume increments of all trees (iv_tree_; m^3^) in the stand irrespective of their species.

6 $$IV_{stand} = \mathop \sum \limits_{stand} iv_{tree} \cdot w_{tree}$$

where, *w*_*tree*_ (unitless) is the statistical weight of each tree within a plot as a result of the tree’s selection area and proximity to its border, and *iv*_*tree*_(m^3^ per 5-years) is the 5-year tree-level volume increment. The variable *iv*_*tree*_ is not provided by the NFI and was, therefore, reconstructed from data on the tree volume (*v*_*tree*_; m^3^), tree radius (*r*_*tree*_; m) and tree radius increment (*ir*_*tree*_; m per 5-years) all three reported by IGN, combined with bark ratios (*β*; unitless) from a French measurement campaign [85]. For this, relations (*f*) between reported tree volume (v_tree_; m^3^) and tree radius (*r*_*tree*_; cm) were fitted independently for each species (Eq. 8) yielding current and previous time-steps tree volume estimates ($\tilde{v}_{tree\left( t \right)} ,\tilde{v}_{{tree\left( {t - 5} \right)}}$; m^3^) from reported current radius (r_tree(t)_; m) and reconstructed previous time-step radius ($\tilde{r}_{{tree\left( {t - 5} \right)}}$; m) all considered above-bark (Eqs. 8 and 9). Previous time-step above-bark radius was calculated from inventory-reported current radius (*r*_*tree*(*t*)_; m), below-bark radius increment ($\widetilde{ir}_{tree\left( t \right)}$; m per 5-years) and bark ratios (β) (Eq. 10).

7 $$iv_{tree} = v_{tree} \left( t \right)\left( {1 - \frac{{\tilde{v}_{{tree\left( {t - 5} \right)}} }}{{\tilde{v}_{tree\left( t \right)} }}} \right)$$

8 $$v_{tree\left( t \right)} = f\left( {r_{tree} } \right) + \varepsilon = \tilde{v}_{tree\left( t \right)} + \varepsilon$$

9 $$\tilde{v}_{{tree\left( {t - 5} \right)}} = f\left( {\tilde{r}_{{tree\left( {t - 5} \right)}} } \right)$$

10 $$r_{{tree\left( {t - 5} \right)}} = \frac{{\left( {1 - \beta } \right)r_{tree\left( t \right)} - ir_{tree\left( t \right)} }}{1 - \beta }$$

For the simulation of diameter–related variables (quadratic mean diameter, diameter growth and stand relative density), it was assumed that each stand could be simulated by the replication of an ‘idealized average tree’ of the main species. The characteristics of the idealized average tree of the stand were based on the observed characteristics of all the trees of this species in the stand. The stand-level radius increment (*Ir*_*stand*_) was hence calculated as the average radius increment of all trees of the main species only (*ir*_*treeSp*_), as reported by the inventory, to account for diversity of growth strategies between different species in mixed stands.

11 $$Ir_{stand} = \frac{{\mathop \sum \nolimits_{stand} ir_{treeSp} \cdot w_{treeSp} }}{{N_{treeSp} }} = \frac{{\mathop \sum \nolimits_{stand} ir_{treeSp} \cdot w_{treeSp} }}{{\mathop \sum \nolimits_{stand} w_{treeSp} }}$$

where *Ir*_*stand*_ is the 5-year radius increment of the idealized average tree replicated in the stand (m), *ir*_*treeSp*_ and *w*_*treeSp*_ are individual tree radius increment (m) and weighting coefficient (unitless), respectively, as given in the NFI data.

The quadratic mean diameter on which harvest decision is based is also calculated from the average basal area for trees of the main species with *g*_*treeSp*_ (m^2^) the individual basal area of trees of the main species and *w*_*treeSp*_ their statistical weights (unitless).

12 $$Dg_{avgtree} = 100\sqrt {\frac{4}{\pi }\frac{{G_{Sp} }}{{N_{Sp} }}} = 100\sqrt {\frac{4}{\pi }\frac{{\mathop \sum \nolimits_{stand} g_{treeSp} }}{{\mathop \sum \nolimits_{Stand} w_{treeSp} }}}$$

where the subscript *Sp* refers to the subset of the stand trees being of the main species. *Dg*_*avgtree*_ is the quadratic mean diameter of the idealized average tree of the stand, G_Sp_ and N_Sp_ are the basal area (m^2^) and number of stems of the trees of the stand’s main species (unitless).

The idealized average tree is then replicated to make up an idealized stand for which the volume is identical to the volume reported in the NFI, providing a reconstructed number of stems in the stand N_avgtree_ used for the calculation of the relative density index.

13 $$v_{avgtree} = \frac{{V_{sp} }}{{N_{sp} }} = \frac{{\mathop \sum \nolimits_{stand} v_{treeSp} \cdot w_{tree} sp}}{{\mathop \sum \nolimits_{stand} w_{treeSp} }}$$

14 $$N_{avgtree} = \frac{{V_{stand} }}{{v_{avgtree} }}$$

where v_avgtree_ is the volume of the idealized average tree, V_Sp_ and N_Sp_ are the total volume (m^3^) and number of stems of the stand’s main species (unitless), respectively, and v_treeSp_ and w_treeSp_ are individual tree volume (m^3^) and statistical weighting coefficient (unitless), respectively. The latter variable describes the representativeness of the tree within the plot, as given in the NFI data.

#### Mortality

No relationship was found in the national inventory data between mortality on the one hand, and age, standing volume and density index on the other hand. Hence, species-specific average mortality rates derived from the national inventory data were applied to all plots as a yearly percentage of standing volume irrespective of plot age. The species-specific mortality rate was defined as the average dead to living volume recorded in the inventory data. Hence, the growth of a plot is the result of the balance between 5-year volume increment and 5-year mortality. After a given age, if a plot’s mortality volume is above its volume increment, then the stand’s volume decreases accordingly. Dead organic matter and soil carbon pools are not included in the model due to the large uncertainties on the effect of management on these carbon stocks and expected minimal differences they would bring between our scenarios.

#### Stand replacement

According to the inventory methodology, trees with diameters below 7.5 cm in diameter are not inventoried [49]. The stands where such trees dominate have not been included for deriving the equations of the growth and harvest simulator, which are hence not valid for very young stands. The first 5 years of post-harvest growth are therefore not simulated by the growth simulator. When stands of more than 200 trees were classified, and trees aged less than 10-years old were reported, but identified as being below the inventory threshold, their per species characteristics were averaged and assumed for the first post-harvest time-step. Data for stands classified as below the inventory threshold and containing less than 200 trees were assumed representative of the recently harvested stands rather than the regrowth and hence were not considered [49]. For species where no data was available according to this constraint, post-harvest stands were replaced by a hypothetical regrowth stand with characteristics of the across species average diameter and volume. Following a harvest, the density and fertility indices of the newly established stand were considered constant as proxies of fertility and management.

#### Thinning and clearcutting

For each species a thinning ratio was defined based on yield tables [86]. The standing volume given by this ratio was subtracted each year from all plots which were subject to thinning, i.e., the actively managed stands under BaU. Thinning affects only stand volume because in our growth and harvest simulator the diameter is not updated. A diameter criterion determined by a species-specific segmented regression was used to decide the final harvest. In the scenarios mobilizing biomass from harvest-delayed stands, the mobilization objective was gradually implemented over the length of the simulation (30 years) following a linear trend.

#### Calibration

The harvested volume of a given species largely depends on the distribution of its stands across the four management approaches and of the yearly thinning ratio. Hence, an initial species-specific mismatch between observed and simulated harvest volumes could be decreased by calibrating the prior thresholds defining the different management approaches. Management approaches are defined by thresholds for exploitability, density index, and harvest diameter.

The threshold on exploitability index was the first to be calibrated as it makes the strongest and most direct impact on harvested volume. Indeed a stand in which exploitability is below the exploitability threshold is simply not managed. As a prior, all stands with exploitability index ‘impossible’ were classified as unexploitable. After iterative comparison of simulated and reported harvest levels, the high harvest levels of *P. pinaster* lead to none of these stands being assigned to the unexploitable category. This result is probably because, the well-developed marketing opportunities for *P. pinaster* incentivize harvest, even when the terrain is sub-optimal.

The two thinning-related parameters, i.e., threshold DI and thinning ratio, have similar effects (Additional file 10: Table S2). The threshold DI for defining the overstocked stands can be raised to decrease the number of overstocked stands, hence increasing the total harvested biomass. Yet, the fraction of biomass removed from thinning at each time-step can also be increased to increase the harvest. Because the prior values of the thinning ratios were based on fragmented data, we believe they come with larger uncertainties than the priors for density index. The priors for thinning ratio were therefore used for optimization against the reported harvest data. This procedure led to *Q. petraea*, *P. abies* and *P. pinaster* thinning ratios being increased from 5, 5 and 10% to 8, 11 and 13%, respectively.

The harvest diameter, above which stands are harvested, was not calibrated (Additional file 10: Table S2). The prior value was thus used for all species. Indeed, this parameter was found to have a rather limited but complex impact on harvested volume because it acted on two different processes in the growth and harvest simulator. First, by setting the threshold for harvest-delayed plots, the harvest diameter controls whether a given plot will be harvested or not. Second, it also defines when all plots under management will be harvested. Hence, depending on the actual diameter distributions of the stands, an increase in the clearcut diameter can increase or decrease the total harvested volume for a given species.

#### Intensifying forest management

The objective of intensifying current forest management is to reach the national energy target for bioenergy production from woody biomass. The simulation experiments applied in this study intensified current forest management by considering three different approaches: decreasing the harvest diameter of actively managed plots, thinning currently overstocked plots and harvesting currently harvest-delayed plots. Each approach entails a family of 10 simulations, which represent the intensity of a given policy targeting: thinning 10 to 100% of the overstocked forest area (scenarios O_v_1 to O_v_10), harvesting 10 to 100% of the harvest-delayed forest area (scenarios D1 to D10), reducing the clearcut diameter of all actively managed forests by 1 to 10 cm (M1–M10).

When the scenario is set to mobilize only a fraction of the targeted forest, the mobilized plots are the ones with the largest diameters (for harvest-delayed forests) or with the highest density (for overstocked forests). Whatever the degree of intensification, the extra mobilization is reached progressively within 30 years by linearly increasing the number of plots targeted from zero to the target area.

### Wood-use modelling

**Fig. 6:**
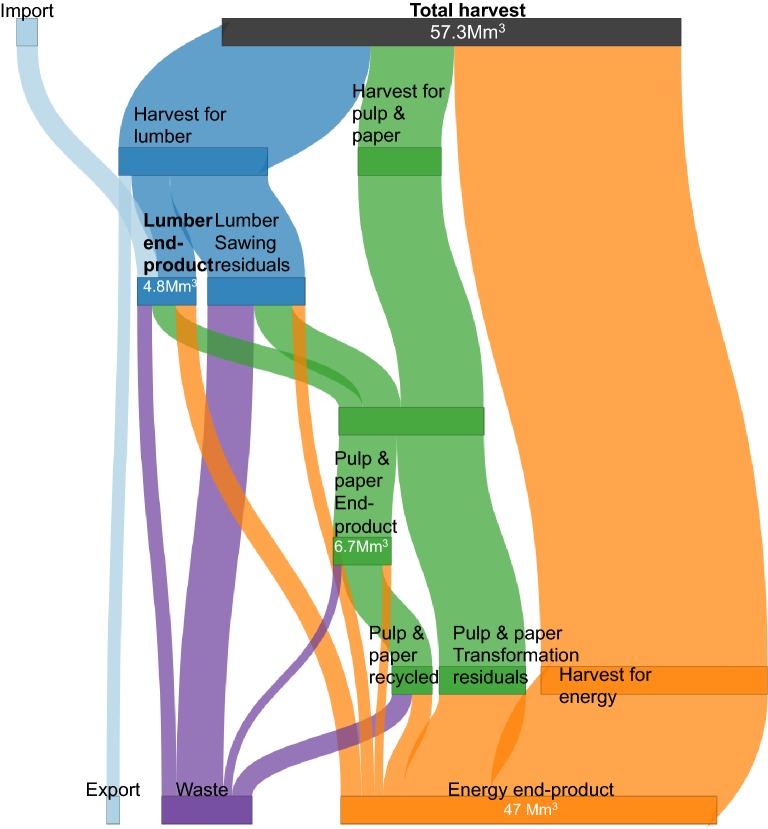
Reconstruction of the main biomass flows in the French wood-use chain from commercial volume harvested to product end-of-life

Wood use substantially contributes to the net carbon balance of French forests, because different wood uses come with different lifetimes, shares of wood reuse and potential substitution of fossil fuel emissions [87]. Therefore, wood flows from the forest to the final product including recycling and the products’ end-of-life were reconstructed. To simplify the scheme, recycling was assumed to occur only once. Data were extracted from different literature sources [16, 88–96] for the year 2010 and reconciled into a unified scheme (Fig. 6).

The wood product scheme starts by assigning harvest to either the lumber or the pulp and energy pathway. The proportions of soft and hardwood going down the timber, pulp and energy pathways were calibrated to match the reported national production of 18.6, 10.4 and 28.3 Mm^3^ including self-consumption energy-wood, respectively [90]. For hardwood, 16%, 14% and 70% of the harvest, were directed to the timber, pulp and industry, and energy pathways, respectively. For softwood, 53%, 23% and 24% of the harvest were directed to the timber, pulp and industry, and energy pathways, respectively. The assignment of harvest from different management practices to timber or pulp and energy was also adjusted to match the reported national values, assuming that harvest from high stand clearcut is preferentially used as timber, whereas the wood from thinning and coppicing is preferentially used in the production of pulp and energy. For softwood, all clearcut and 26% of thinnings were directed to timber, and 74% of thinnings were directed to pulp and paper. For hardwood, all thinnings and 43% of clearcuts were directed down the pulp and energy pathway, and 57% of the clearcuts were directed to timber.

### Carbon balance

The carbon balance is calculated by summing all components of the life cycle of wood: sequestration in the forest, carbon storage in wood products, carbon emission at the end of the product’s life and the avoided emissions from substitution. Each wood use is assigned an average life expectancy and substitution potential (Table 4). The BaU scenario is used as the reference for substitution calculation hence assuming that literature-based substitution coefficients used were derived for reference and scenario conditions consistent with the wood harvest and wood-use practices implicitly described in our scenarios. The avoided emissions under each scenario were calculated by multiplying the extra wood products with respect to BaU by their respective substitution coefficients.

Carbon storage in wood products is only accounted for in the first wood use (timber end-product, pulp and paper end-product or energy) and the carbon contained in the products is emitted progressively at each time-step following an exponential decay function. However, for substitution effects, consecutive wood uses are accounted for by multiplying their respective volumes with product-specific substitution coefficients all supposed to occur at time of harvest. This assumption is considered to have a minor effect on the results since most consecutive wood uses occur in a time lapse below the 5-year time-step of the forest growth and harvest simulator. This time compression approach leads to a small overestimation of substitution potential of harvest directed to timber with the anticipation of the substitution benefits from product recycling.

## Additional files

**Additional file 1.** Continuation of present-day forest management.

**Additional file 2: Figure S1.** Total harvested commercial volume per year (Mtoe/yr) from the three intensification scenarios separately (D, O_v_, and M) and all three scenarios combined (D+ O_v_ +M) between 2015 and 2040. Blue shows all three scenarios combined (D+ O_v_ +M), brown shows intensification of actively managed sites (M), red shows intensification of harvest-delayed sites (D), green shows intensification of overstocked sites (O_v_), and black shows business as usual management of all sites (BaU).

**Additional file 3: Figure S2.** Projected evolution of the French forest sector carbon balance for the three intensification scenarios separately (D, O_v_, and M) and all three scenarios combined (D+ O_v_ +M) between 2010 and 2115. Blue shows all three scenarios combined (D+ O_v_ +M), brown shows intensification of actively managed sites (M), red shows intensification of harvest-delayed sites (D), green shows intensification of overstocked sites (O_v_), and black shows business as usual management of all sites (BaU). The intensification scenarios are presented in section “Methods”—Intensifying forest management and summarized in Table 3.

**Additional file 4: Figure S3.** Comparison of the observed (grey) and estimated (black) production characteristics per species (a, c, e) and per wood type (b, d, f). (a-b) standing volume in 2010, (c-d) annual biological production between 2010 and 2015, and (e-f) annual harvest estimated between 2008 and 2012.

**Additional file 5: Figure S4.** Model parameterized and inventory-reported standing volume (m^3^), (b) biological production (m^3^ yr^−1^) per species and region. Green points refer to hardwood species and purple points refer to softwood species.

**Additional file 6: Table S3.** Site and density indices fitted parameters.

**Additional file 7: Figure S5.** Derivation of site indices for *Quercus robur* stands. The bold line represents the guide curve fitted for all plots assumed to be high stands (green points). Thin lines are sample curves derived from the guide curve to project height at 100 years for sample stands.

**Additional file 8: Figure S6.** Derivation of density indices for *Quercus robur* stands. The self-thinning line (red) is derived as the statistical envelope of the green point cloud comprised of those points between the 10^th^ and 90^th^ age percentiles. The resulting distribution of density indices is shown in the upper right histogram.

**Additional file 9: Table S1.** Exploitability indices adapted from IGN methodology [49 Colours indicate the level of exploitability from easy (green), medium (yellow), difficult (orange) to impossible (brown).

**Additional file 10: Table S2.** Current practice parameters for harvest and thinning.

## Abbreviations

BaU: business as usual
(M)ha: (million) hectare
(M)toe: (million) tons of oil equivalent
NFI: National Forest Inventory
