# Supplementary material for: Carbon costs and benefits of France’s biomass energy production targets
Source: Carbon Balance Manag. 2018 Dec 13;13:26. doi: 10.1186/s13021-018-0113-5 (PMC6292836; doi:10.1186/s13021-018-0113-5)
Supplement: Supplementary file 1 — Additional file 1. Continuation of present-day forest management. [file 13021_2018_113_MOESM1_ESM.docx]

S1. Continuation of present-day forest management

**Present-day wood production**

Because the different management approaches are interdependent, calibration could not perfectly match the observed harvest (see Limitations). Matching initial model values and inventory-reported data were interpreted as an indication of the validity of the resource description assumptions and credibility of the model for future projections. Over all tree species at the national scale, the relative error for standing volume, weighted per species area, amounts to an average of 19%, and when aggregated by wood type only, relative errors for standing volume at national scale were 7% for hardwood and <1% for softwood (Fig. S3b).

At species level, the relative error of the initial standing volume at national scale ranges from <1% for Pinus sylvestris to 56% for other softwood (Fig. S3a), whereas data grouped per species and region had an r^2^ value of 0.97 (Fig. S3a). The initial biological production of forest directly results from the modelling assumptions and therefore could be used to evaluate the model’s ability to describe the resource (Fig. S3c-d). The relative error per species weighted per species area amounts to 29% over all species at the national scale (Fig. S3c), and when aggregated by wood type, the relative error went down to 12 and 17% for softwood and hardwood, respectively (Fig. S3d). At the species scale, the relative error ranged from 4% for Abies alba to 68% for Quercus robur (Fig. S3c) whereas data grouped only per species and region had an r2 value of 0.87 (Fig. S3b). For harvested wood, the relative error between the model and the inventory data per species ranged from <1 % for *Quercus robur* to 63% for *other softwood* (Fig. S3e) with an area weighted average relative error of 20% (Fig. S3c). When grouped by wood type, the relative error amounts to 1% and 9% for hardwood and softwood, respectively (Fig. S3f).

**Present-day wood use**

Wood flows in the French timber industry were reconstructed from data that were aggregated by wood type (see Methods – Wood-use modelling). The reconstruction ranges from harvest to end-of-life of the wood products and covers the years 2010 to 2015. Such a reconstruction comes with substantial (>50%) uncertainties for every step in the wood-use chain due to inconsistencies between different data sources and substantial uncertainties of individual data sources.

All species and management combined, 32% of the total harvest was found to be directed to the timber industry (53% of softwood harvest and 16% for hardwood harvest), 18% to the pulp and paper industry (24% of softwood harvest and 13% of hardwood harvest) and 50% to be used as biofuel (23% of softwood harvest and 71% of hardwood harvest) (Fig. 6). For a given wood type, the fraction of thinnings and clearcut used for timber, pulp or bioenergy were adjusted to match the reported values. For hardwood, pulp and energy were found to be the destination of all thinnings and 43% of the clearcut, while the remaining 57% of the clearcut is directed to timber. For softwood, timber was evaluated be made of all the clearcut wood and 26% of the thinnings while 74% of the thinnings are directed to pulp and energy.

Cascading wood use and recycling increases the time that carbon spends in the wood chain and thus out of the atmosphere and should, therefore, be accounted for when estimating the carbon balance of the forestry sector. At each step of the chain, residue is generated which is directed towards transformation processes that can handle wood of lower quality and/or smaller dimensions. For example, according to our reconstruction, the sawing of high-grade wood generates 65% and 50% residues for hardwood and softwood, respectively. Thirty nine percent of these residues are then used for chipboard and pulp production, 13% for bioenergy and 48% is considered waste.

Likewise in France, only 25% of the timber is estimated to end its life in landfills, 35% to be used as biofuel and 40% recycled into industrial or pulp wood products (Fig. 6). Hence, as a consequence of increasing the harvest of high-grade timber more biomass would become available for use as biofuel. Overall, our reconstruction indicates that currently 40% of the wood energy originates from cascading wood use, either from burning wood products at their end-of-life or by using residuals produced along the wood transformation chain (Fig. 6).

**Future wood production and wood use under BaU**

Under BaU, the growth and harvest simulator projects an 11% decrease in the biological production from 87 Mm^3^ yr^-1^ in 2010 down to 78 Mm^3^ yr^-1^ in 2040, resulting from a large number of stands reaching maturity. On the one hand the unexploitable and harvest-delayed stands see their mean age increase from 89 to 114 years old, associated with a reduction of their mean annual volume increment per unit area from 4.6 to 5 m^3^ ha^-1^ yr^-1^. On the other hand, in the same 30 years, the average age of the actively managed and overstocked stands only increased by 14 years due to 78% of the actively managed and overstocked stands having been clearcut. This change in age structure resulted in a decrease of the mean annual volume increment of the actively managed and overstocked stands from 6.5 to 6.1 m^3^ ha^-1^ yr^-1^. This decrease in biological production along with the increase of harvest from actively managed stands from 42 Mm^3^ yr^-1^ in 2015 to 49 Mm^3^ yr^-1^ in 2040 would result in the overall harvest ratio for France increasing from 50% in 2010 to 63% in 2040. Nevertheless, annual wood increment would remain well above harvest, contributing to 273 Tg of carbon being stored in the in-situ forest carbon pools between 2010 and 2040.

Applying a BaU scenario to the wood-use chain and assuming flexibility of the wood industry in terms of processing capacity, the age structure evolution of French forest (see S3) would increase timber production by 24%, pulp and paper production by 17%, and wood-based biofuel by 9% by 2040. Bioenergy production is thus projected to increase under a BaU scenario, but would still fall 14 to 19% short of meeting the national energy production targeted range in 2025 (Fig. 2). The increased production of wood products is projected to be dominated by long-living timber products which would in turn increase the carbon stock in wood products by 122 Tg C. When accounting for both carbon storage in forests and wood products, the BaU scenario would thus increase the carbon storage of the French forestry sector by 396 Tg C by 2040.
