# Supplementary figures and images for: Carbon costs and benefits of France’s biomass energy production targets
Source: Carbon Balance Manag. 2018 Dec 13;13:26. doi: 10.1186/s13021-018-0113-5 (PMC6292836; doi:10.1186/s13021-018-0113-5)

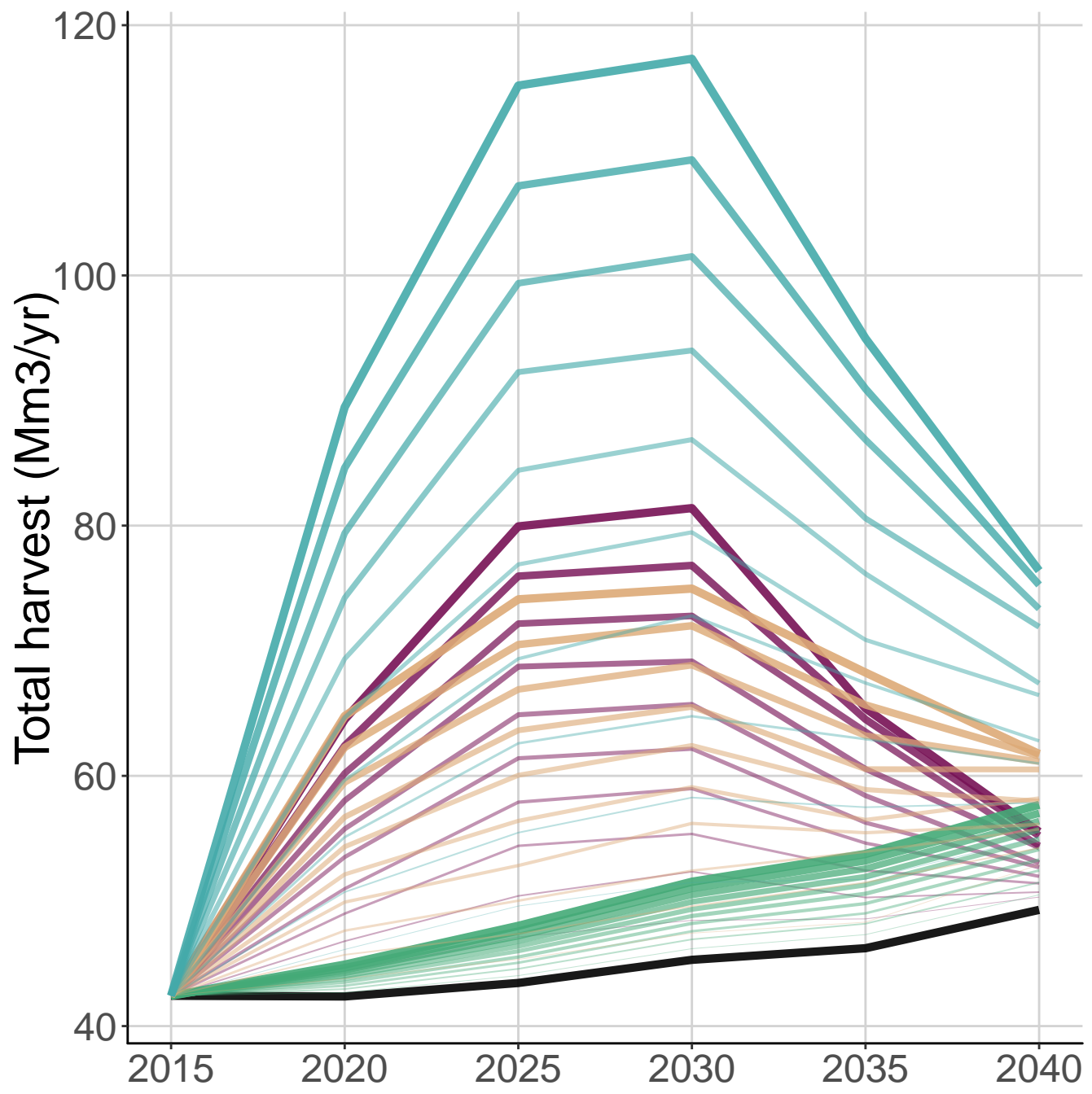

Supplement: Supplementary file 2 — Additional file 2: Figure S1. Total harvested commercial volume per year (Mtoe/yr) from the three intensification scenarios separately (D, Ov, and M) and all three scenarios combined (D+ Ov +M) between 2015 and 2040. Blue shows all three scenarios combined (D+ Ov +M), brown shows intensification of actively managed sites (M), red shows intensification of harvest-delayed sites (D), green shows intensification of overstocked sites (Ov), and black shows business as usual management of all sites (BaU). [file 13021_2018_113_MOESM2_ESM.pdf]

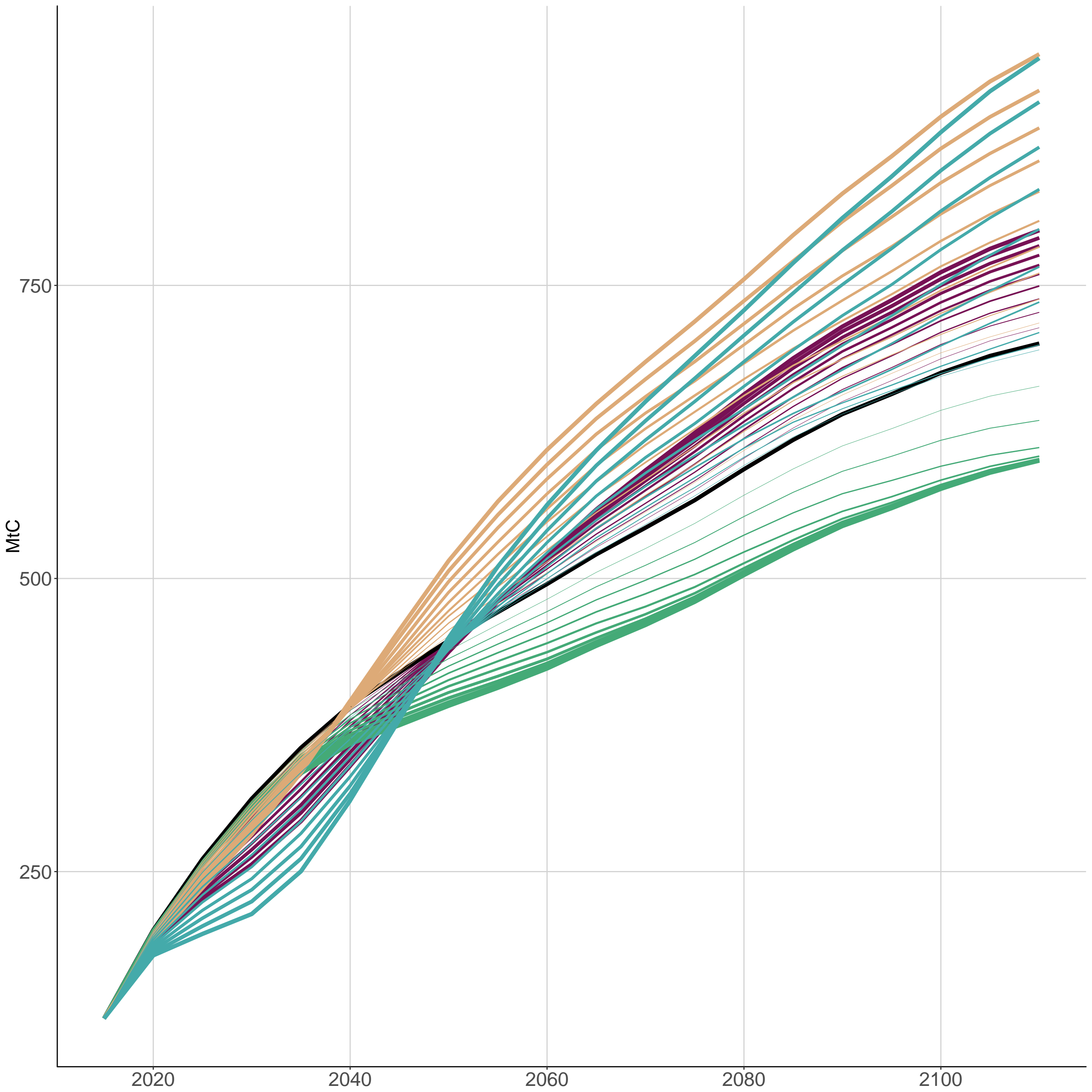

Supplement: Supplementary file 3 — Additional file 3: Figure S2. Projected evolution of the French forest sector carbon balance for the three intensification scenarios separately (D, Ov, and M) and all three scenarios combined (D+ Ov +M) between 2010 and 2115. Blue shows all three scenarios combined (D+ Ov +M), brown shows intensification of actively managed sites (M), red shows intensification of harvest-delayed sites (D), green shows intensification of overstocked sites (Ov), and black shows business as usual management of all sites (BaU). The intensification scenarios are presented in section “Methods”—Intensifying forest management and summarized in Table 3. [file 13021_2018_113_MOESM3_ESM.pdf]

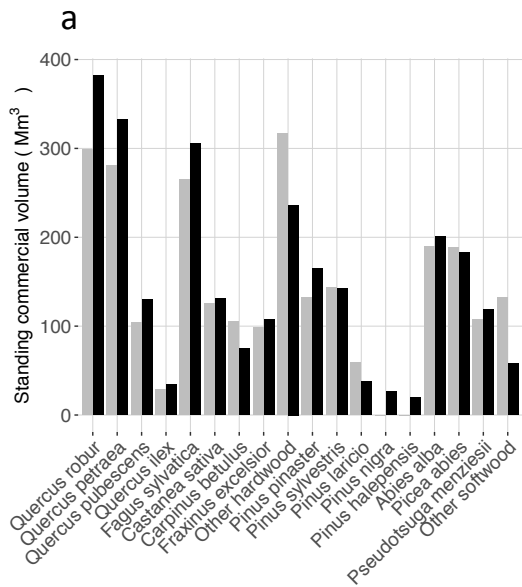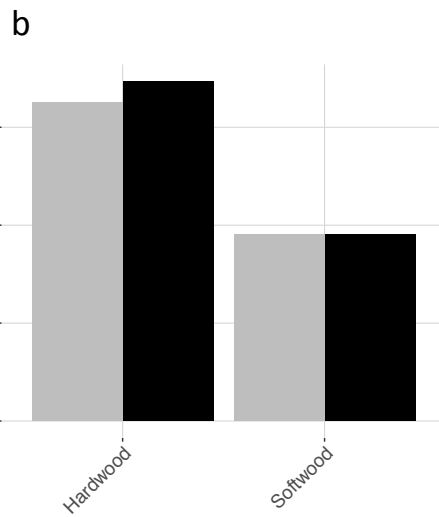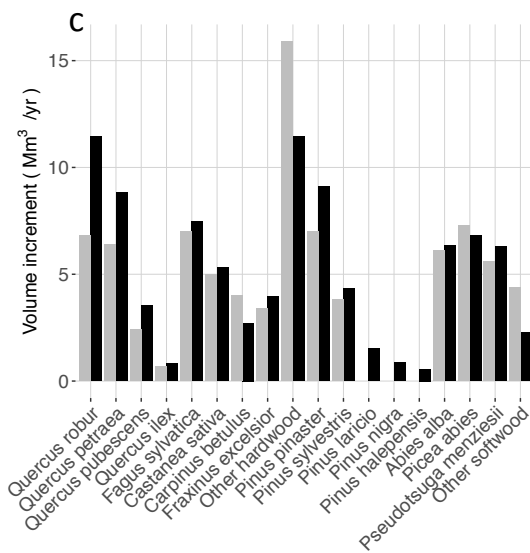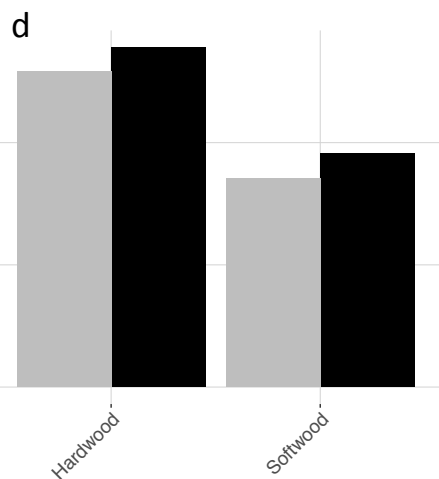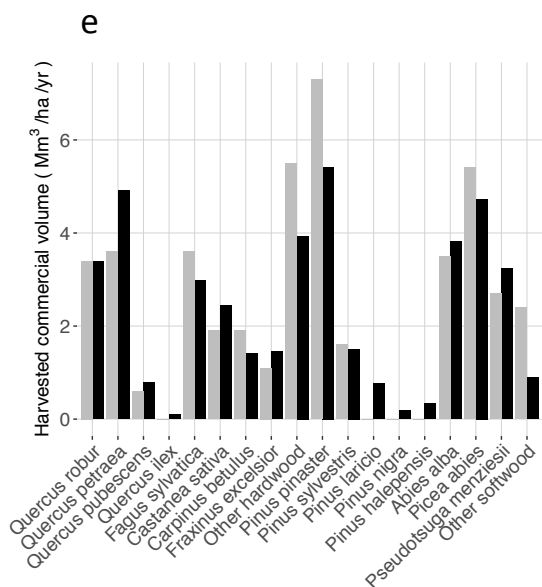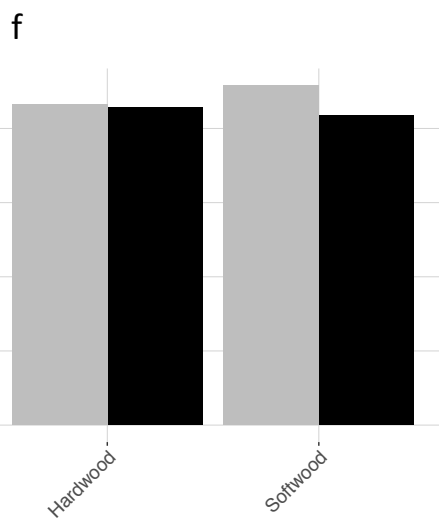

Supplement: Supplementary file 4 — Additional file 4: Figure S3. Comparison of the observed (grey) and estimated (black) production characteristics per species (a, c, e) and per wood type (b, d, f). (a-b) standing volume in 2010, (c-d) annual biological production between 2010 and 2015, and (e-f) annual harvest estimated between 2008 and 2012. [file 13021_2018_113_MOESM4_ESM.pdf]

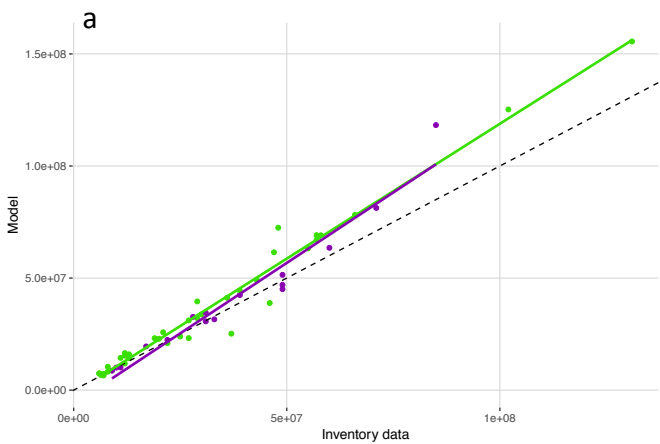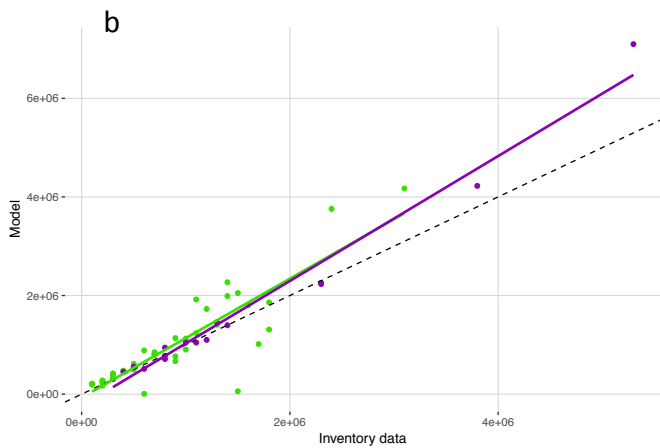

Supplement: Supplementary file 5 — Additional file 5: Figure S4. Model parameterized and inventory-reported standing volume (m3), (b) biological production (m3 yr−1) per species and region. Green points refer to hardwood species and purple points refer to softwood species. [file 13021_2018_113_MOESM5_ESM.pdf]

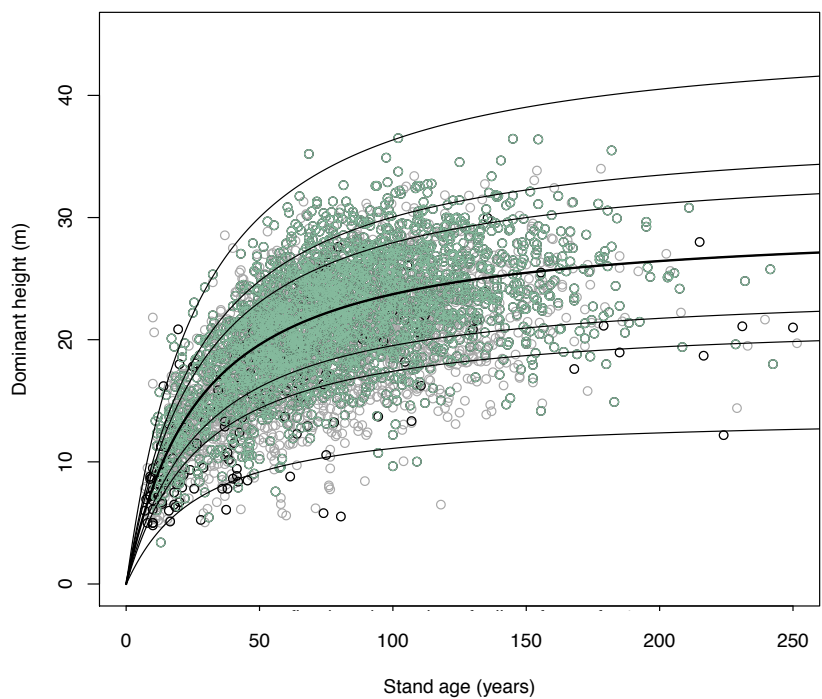

Supplement: Supplementary file 7 — Additional file 7: Figure S5. Derivation of site indices for Quercus robur stands. The bold line represents the guide curve fitted for all plots assumed to be high stands (green points). Thin lines are sample curves derived from the guide curve to project height at 100 years for sample stands. [file 13021_2018_113_MOESM7_ESM.pdf]

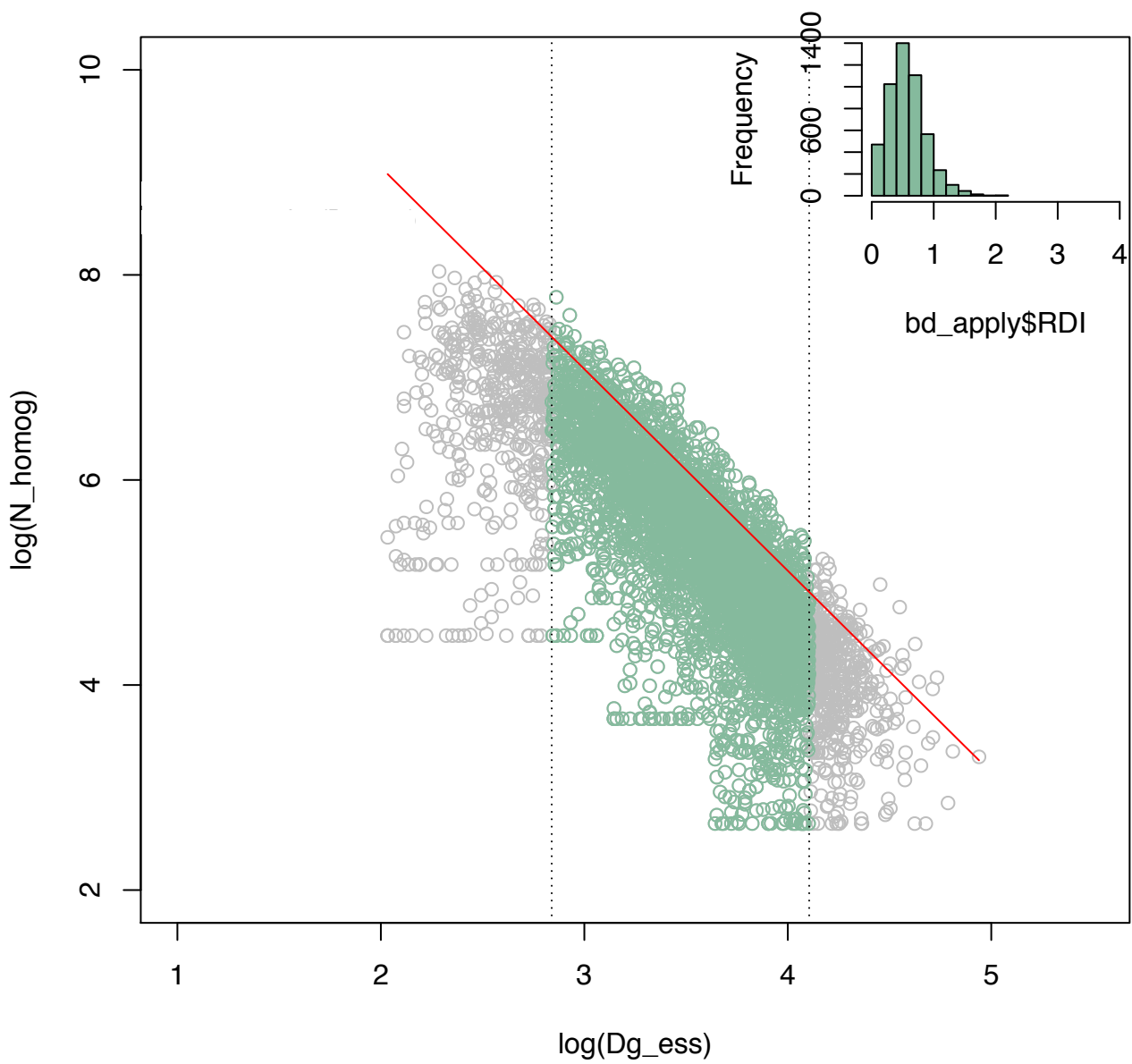

Supplement: Supplementary file 8 — Additional file 8: Figure S6. Derivation of density indices for Quercus robur stands. The self-thinning line (red) is derived as the statistical envelope of the green point cloud comprised of those points between the 10th and 90th age percentiles. The resulting distribution of density indices is shown in the upper right histogram. [file 13021_2018_113_MOESM8_ESM.pdf]
